# Viral Respiratory Epidemic Modeling of Societal Segregation Based on Vaccination Status

**DOI:** 10.7759/cureus.50520

**Published:** 2023-12-14

**Authors:** Joseph Hickey, Denis G Rancourt

**Affiliations:** 1 Research, Correlation Research in the Public Interest, Ottawa, CAN

**Keywords:** quarantine, covid-19 isolation, contact frequency, sir model, vaccination-status-based segregation, vaccination mandate, vaccinated, unvaccinated, covid 19, vaccine passports

## Abstract

Background: Societal segregation of unvaccinated people from public spaces has been a novel and controversial coronavirus disease 2019 (COVID-19)-era public health practice in many countries. Models exploring potential consequences of vaccination-status-based segregation have not considered how segregation influences the contact frequencies in the segregated groups. We systematically investigate implementing effects of segregation on population-specific contact frequencies and show this critically determines the predicted epidemiological outcomes, focusing on the attack rates in the vaccinated and unvaccinated populations and the share of infections among vaccinated people that were due to contacts with infectious unvaccinated people.

Methods: We describe a susceptible-infectious-recovered (SIR) two-population model for vaccinated and unvaccinated groups of individuals that transmit an infectious disease by person-to-person contact. The degree of segregation of the two groups, ranging from zero to complete segregation, is implemented using the like-to-like mixing approach developed for sexually transmitted diseases, adapted for presumed severe acute respiratory syndrome coronavirus 2 (SARS‑CoV‑2) transmission. We allow the contact frequencies for individuals in the two groups to be different and depend, with variable strength, on the degree of segregation.

Results: Segregation can either increase or decrease the attack rate among the vaccinated, depending on the type of segregation (isolating or compounding), and the contagiousness of the disease. For diseases with low contagiousness, segregation can cause an attack rate in the vaccinated, which does not occur without segregation.

Interpretation: There is no predicted blanket epidemiological advantage to segregation, either for the vaccinated or the unvaccinated. Negative epidemiological consequences can occur for both groups.

## Introduction

Models can be used to investigate infectious disease dynamics under different hypotheses about the characteristics of a disease and the effects of health policy. In this endeavour, there are advantages to working with the simplest possible but sufficiently realistic models [1,2], where one should exclude simple models that are not sufficiently realistic for the intended application, either because of their structure or because of incorrect assumptions about the underlying mechanisms. Following this approach, researchers have extended the foundational simple susceptible-infectious-recovered (SIR)-type model to explore diseases with birth and death dynamics, maternal- or vaccine-derived immunity, latency of infection, patterns of contact mixing between different societal groups, and so on [3-7], and to study the effect of isolating vulnerable individuals from the general population during a pandemic, in the absence of vaccination [8].

Recently, SIR models of epidemic dynamics have been implemented with two interacting societal groups (vaccinated and unvaccinated) to examine epidemic outcomes for variable degrees of interaction between the two groups, including whether the unvaccinated put the vaccinated unduly or disproportionately at risk, using epidemiological parameters intended to be representative of severe acute respiratory syndrome coronavirus 2 (SARS‑CoV‑2) [9-12]. These prior implementations regarding groups differentiated by vaccination status take the contact frequencies of the majority and socially excluded groups to be equal and held constant, irrespective of the degree of segregation (or exclusion or “like-to-like mixing”), which is not realistic.

Here, we implement population-specific contact frequencies that can be different for the two groups and can either increase or decrease with increasing segregation. This is necessary because, for example, in many actual regulatory policies, the excluded unvaccinated group is barred from public venues or services where people gather and from public transport where people are in close proximity for various durations. In general, the contact frequency of the excluded group decreases with increasing segregation if isolation is in effect, and increases with increasing segregation if the excluded individuals are crowded together. Implementing this essential model feature gives rise to the more complex behaviour of the attack rates in the vaccinated and unvaccinated populations (*A_v_* and *A_u_*, respectively), which can increase or decrease, or rise to a maximum before decreasing, as the two groups are increasingly segregated. This is also true for the share (*B_v_*) of infections among vaccinated people that are due to contact with infectious unvaccinated people.

This article was previously posted to the medRxiv preprint server on August 23, 2022, and revised versions were posted to medRxiv on November 28, 2022, July 6, 2023, July 19, 2023, and October 31, 2023.

## Materials and methods

Model design

We adopt the standard SIR framework in a structure with two sub-populations. If a susceptible person (S) comes into contact with an infectious person (I), the susceptible person can become infectious, and infectious people eventually recover (R) and become permanently immune.

We divide the population into two groups: vaccinated and unvaccinated. Vaccination is “all or nothing”, such that a proportion, *VE, *of the vaccinated population is immune (is in the R state from the outset of the simulation), where the parameter *VE *represents vaccine efficacy. The model also includes a natural immunity parameter, *NI*, equal to the proportion of unvaccinated that are immune from the outset due to previous infection [9].

The model consists of the following six differential equations: 

$$\frac{dS_u}{dt} = -c_u\beta_uS_u\left[f_{uv}\frac{I_v}{N_v} + f_{uu}\frac{I_u}{N_u} \right ]$$

$$\frac{dI_u}{dt} = c_u\beta_uS_u\left[f_{uv}\frac{I_v}{N_v} + f_{uu}\frac{I_u}{N_u} \right ] - \gamma_u I_u$$

$$\frac{dR_u}{dt} = \gamma_u I_u$$

$$\frac{dS_v}{dt} = -c_v\beta_vS_v\left[f_{vu}\frac{I_u}{N_u} + f_{vv}\frac{I_v}{N_v} \right]$$

$$\frac{dI_v}{dt} = c_v\beta_vS_v\left[f_{vu}\frac{I_u}{N_u} + f_{vv}\frac{I_v}{N_v} \right] - \gamma_v I_v$$

$$\frac{dR_v}{dt} = \gamma_v I_v$$

*S_u_*, *I_u_*, and *R_u_* represent the number of susceptible, infectious, and recovered unvaccinated people, at time *t*. *N_u_* represents the total number of unvaccinated people. *c_u_* represents the population-specific contact frequency (number of contacts per unit time) of unvaccinated people. β*_u_* is the probability that a susceptible unvaccinated person becomes infected upon contact with an infectious person (regardless of whether the infectious person is vaccinated or unvaccinated). γ_u_ is the rate at which infected unvaccinated people recover from infection. The quantities *S_v_*, *I_v_*, *R_v_*, *N_v_*, *c_v_*, β*_v_*, and γ_v_ are defined equivalently, for vaccinated people.

*f_ij_* is the probability that a person of type *i* (either *u* or *v*) has contact with a person of type *j* (either *u* or *v*) and is modulated by a parameter, η, which controls the degree of segregation between vaccinated and unvaccinated people (see Appendix 1 for technical details). When η = 0, there is no segregation, and the two groups mix randomly. When η = 1, there is complete segregation, such that the vaccinated only come into contact with other vaccinated, and the unvaccinated only come into contact with other unvaccinated.

The parameter η follows from Garnett and Anderson [1], who modelled sexually transmitted disease spread in a population divided into groups with different frequencies of sexual contact. They take the contact frequency to be a constant characteristic of the individuals within a group. However, contact frequency is not generally and solely an intrinsic individual characteristic [13].

In our model, the population-specific contact frequencies of the vaccinated and unvaccinated individuals (*c_v_* and *c_u_*, respectively) can increase, decrease, or remain constant as the two groups are segregated. We implement a new approach to achieve this: we keep the first two terms in Taylor expansions of *c_v_* and *c_u_* versus η (*c_v_* =  (1 + *m_v_*η) and *c_u_* =  (1 + *m_u_*η); see Equations A3 in Appendix 1). Thus, *m_v_* and *m_u_* determine the degree of increase or decrease of the contact frequency in either group, as η is increased.

For example, when *m_u_* < 0, as segregation is increased, the contact frequency of unvaccinated people decreases. This corresponds to a segregation policy that excludes unvaccinated people from public spaces such as restaurants, cinemas, workplaces, airplanes, trains, etc. [14-17]. Conversely, *m_u_* > 0 corresponds to a segregation policy that increases contact between unvaccinated people; for example, by requiring returning unvaccinated travelers to stay in designated facilities [18-20].

In principle, the vaccinated and unvaccinated contact frequencies may be different even when the two groups are completely unsegregated. The unsegregated (η = 0) contact frequencies are set by the parameters $c_v^0$ and $c_u^0$. 

As can be seen from the six differential equations defining our model, there are two “β parameters”, two “*c* parameters” and two “γ parameters” in our model. Since each β parameter always occurs as part of a product with its respective *c* parameter, the β parameters can freely be set equal to 1. We set β_v_ = β_u_ = 1 in this paper, without any loss of generality. This implies that, by definition (since β = 1), the contact frequencies “*c*” in our model are conceptually for contacts that are of sufficiently close proximity and long duration that an infection is guaranteed to occur when a susceptible and an infectious person meet [7,8]. For a more contagious virus, more of an individual’s contacts are long and close enough that transmission would be guaranteed, corresponding to higher $c_v^0$ and $c_u^0$. In other words, setting β_v_ = β_u_ = 1 is equivalent to redefining “*c*” as the product “β*c*”, thus eliminating a redundant parameter, without any loss of generality.

The model of Fisman et al. [9] is the special case of our model with *m_u_* = *m_v_* = 0, $c_v^0\beta_v = c_u^0\beta_u$ and γ*_v_* = γ*_u_* in which case the equal contact frequencies of both vaccinated and unvaccinated remain constant regardless of the level of segregation. Such an implementation does not represent how segregation has been applied during the coronavirus disease 2019 (COVID-19) era in Canada and many countries [14-17,21], since unvaccinated people were excluded from public spaces while vaccinated people were allowed access, thus changing venues and opportunities for contact as segregation is imposed.

Throughout this paper, “contact frequency” refers to the frequency of infectious contacts, since the probability of infection per infectious-susceptible contact is set equal to 1 without loss of generality (see Appendix 1).

Model parameterization

The parameters of our model are listed in Table 1 and calculated quantities are given in Table 2. Technical details of the model are in Appendix 1.

**Table 1:** Model parameters

Parameter description	Symbol	Typical value	Bound
Degree of segregation of vaccinated and unvaccinated groups	η	(varied)	0 to 1
Contact frequency of vaccinated people when η = 0	$c_v^0$	300 contacts/yr	≥ 0
Contact frequency of unvaccinated people when η = 0	$c_u^0$	300 contacts/yr	≥ 0
Probability of transmission per contact between a susceptible vaccinated person and an infected person	β*_v_*	1	0 to 1
Probability of transmission per contact between a susceptible unvaccinated person and an infected person	β_u_	1	0 to 1
Degree of increase ($m_v > 0$) or decrease ($m_v &lt; 0$) of vaccinated contact frequency as a function of η	$m_v$	0	≥ -1
Degree of increase ($m_u > 0$) or decrease ($m_u &lt; 0$) of unvaccinated contact frequency as a function of η	$m_u$	varied	≥ -1
Rate of recovery from infection of a vaccinated person (per year)	γ*_v_*	73 yr^-1^	≥ 0
Rate of recovery from infection of an unvaccinated person (per year)	γ_u_	73 yr^-1^	≥ 0
Population fraction of vaccinated people	P_v_	0.8	0 to 1
Vaccine efficacy	VE	0.8	0 to 1
Proportion of unvaccinated population with natural immunity	NI	0.2	0 to 1
Population of entire society	N	10^7^	> 0

**Table 2:** Quantities calculated from model results Mathematical definitions of the quantities in Table 2 can be found in Appendix 1, Section A1.3.

Name	Symbol
Attack rate in the vaccinated population	A_v_
Attack rate in the unvaccinated population	A_u_
Attack rate in the overall population (vaccinated and unvaccinated)	A_t_
Share of infections among vaccinated people that were due to contacts with infectious unvaccinated people	B_v_

Analysis

The attack rate among the vaccinated population is defined as the proportion of initially susceptible vaccinated people who become infected during the epidemic:

$A_v = \frac{S_v(t_0)-S_v(t_f)}{S_v(t_0)}$

where *S_v_*(t0) is the number of susceptible vaccinated people at the beginning of the epidemic and *S_v_*(*t_f_*) is the number of susceptible vaccinated people remaining once there are no longer any infectious people in the entire (vaccinated and unvaccinated) population. *A_u_* is defined equivalently, for the unvaccinated, replacing the *v* subscripts with *u* in the equation A_v_ above.

The overall attack rate for the full (vaccinated plus unvaccinated population) is:

$A_t = \frac{(S_v(t_0)+S_u(t_0))-(S_v(t_f)+S_u(t_f))}{S_v(t_0)+S_u(t_0)}$

We also define *B_v_* as the share of infections among vaccinated people that were due to contact with infectious unvaccinated people (see Equation A6 of Appendix 1).

We focus on segregation types that are targeted at the unvaccinated group. We assume, for simplicity, that segregation has no impact on the contact frequency of vaccinated people (*m_v_* = 0). We also assume that the contact frequencies in both groups are the same when there is no segregation ($c_v^0\ = c_u^0$). We use the same values as used by Fisman et al. [9] for the remaining parameters: *P_v_* = 0.8, *VE *= 0.8, *NI *= 0.2, γ_v_ = γ*_u_* = 73 yr^-1^, and *N* = 10^7^. These values were intended to be representative of COVID-19 and vaccination; in particular, the recovery rate of 73 yr^-1^ is equivalent to a recovery time of five days [22,23] and is assumed to be the same for vaccinated and unvaccinated people.

Appendix 2 contains supplementary figures with results for different parameter combinations, including *P_v_* ≠ 0.8, *m_v_* ≠ 0 and $c_v^0 \neq c_u^0$. In all results in this paper, simulations were initiated with a seed number of 100 infectious individuals distributed proportionately among the two sub-populations.

## Results

**Figure 1:**
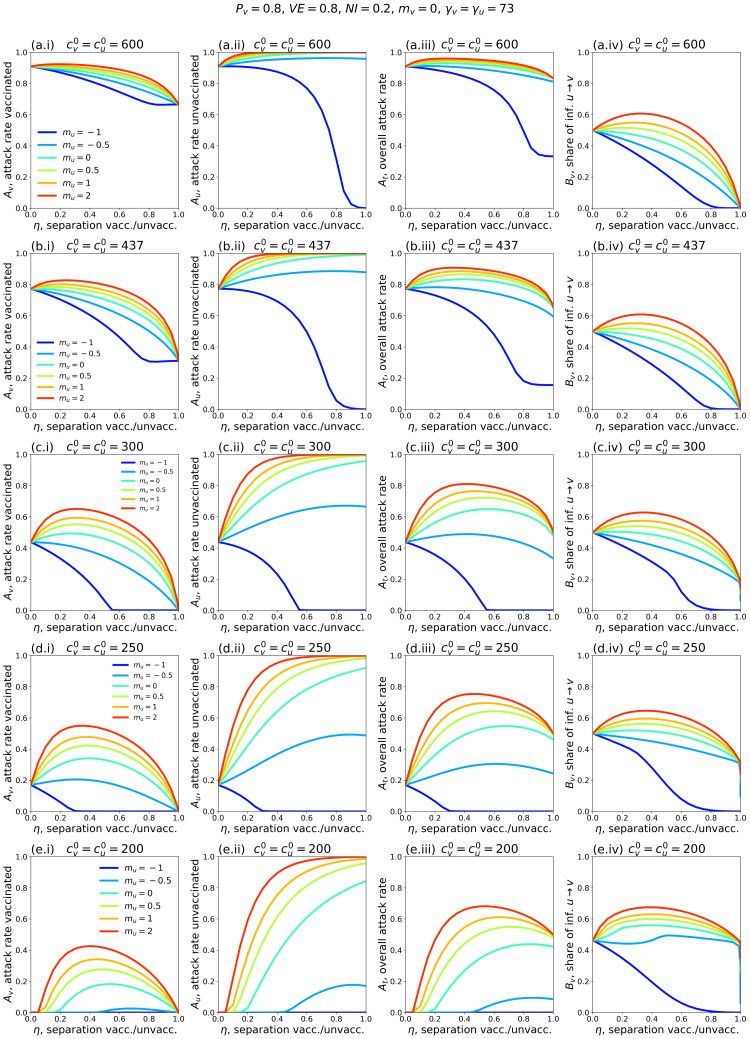
Attack rates as functions of the degree of segregation of vaccinated and unvaccinated populations Attack rates *A_v_* (vaccinated population), *A_u_* (unvaccinated population), *A_t_* (overall population) and share of vaccinated infections that were due to contacts with unvaccinated people, *B_v_*, as functions of the degree of segregation, η, between the vaccinated and unvaccinated. Each row of panels shows *A_v_*, *A_u_*, *A_t_* and *B_v_* for a particular choice of $c_v^0 = c_u^0$. Values of fixed model parameters are indicated at the top of the figure. For reference, in a single-population (no vaccination) model, the corresponding *R*_0_ values for rows a-e of the figure are 8.2, 6.0, 4.1, 3.4 and 2.7, respectively.

Figure 1 shows simulation results for a range of model parameters for different epidemiological conditions and degrees and types of societal segregation. Each row of panels is for a fixed value of $c_v^0 = c_u^0$, which decreases moving from the top row (Figures 1a.i-iv) to the bottom (Figures 1e.i-iv). The left column of panels shows how the attack rate among the vaccinated population, *A_v_*, changes with the degree of segregation, η. The second and third columns show *A_u_* and *A_t_* as functions of η, respectively, and the right column shows how *B_v_*, the share of vaccinated infections that were due to contacts with unvaccinated people, varies with η.

Figures 1c.i-iv show results for a moderate value of contacts per year. For reference, in a single-population (no vaccination) model, $c_v^0 = c_u^0$ = 300 contacts/year and γ_v_ = γ*_u_* = 73 years^-1^ corresponds to a basic reproduction number *R*_0_ = c/γ = 4.1.

In Figure 1c.i, when *m_u_* =-1 and *m_u_* = -0.5 (reflecting large and moderate degrees of exclusion and isolation of unvaccinated people), the vaccinated attack rate, *A_v_* decreases with increasing segregation. However, when *m_u_* > 0 (compounding of unvaccinated people) or *m_u_* = 0 (segregation has no influence on contact frequency of unvaccinated people), there is a maximum in *A_v_* for moderate values of η. Therefore, with compounding segregation, large values of η are required for *A_v_* to be lower than its value for no segregation (η = 0). Figure 1c.ii shows that the unvaccinated attack rate, *A­_u_*, increases with segregation for anything other than strong isolating segregation (*m_u_* approaching -1). This produces a maximum in the overall attack rate, *A_t_*, at moderate degrees of segregation, even for values of *m_u_* for which *A_v_* decreases monotonically (*m_u_* = -0.5). Figure 1c.iv shows that *B­_v_*, the share of vaccinated infections that are due to unvaccinated people, has a shape similar to *A_v_*(η, *m_u_*). In all panels, 20% of the total population is unvaccinated (*P_v_* = 0.8; Table 1).

Figures 1c.i-iv, therefore, demonstrate that whether applying segregation increases or decreases the vaccinated population attack rate depends on both the degree of segregation and how segregation affects contact frequency.

Figures 1a.i-iv and Figures 1b.i-iv show results for larger $c_v^0 = c_u^0$. Compared to Figure 1c.i, in Figure 1a.i and Figure 1b.i, *A_v_* does not increase much with η when *m_u_* > 0, and *A_v_* no longer has a maximum when *m_u_* = 0. It can also be seen that *A_v_* increases with increasing $c_v^0 = c_u^0$ when there is no segregation (η = 0).

Reducing $c_v^0 = c_u^0$ (Figures 1d.i-iv and Figures 1e.i-iv) decreases *A_v_*(η = 0), and larger η can dramatically increase *A_v_*. Even with an isolating segregation policy (*m_u_* = −0.5 in Figure 1d.i), *A_v_* is increased for moderate values of η.

When $c_v^0 = c_u^0$ are small enough, ($c_v^0 = c_u^0$ = 200 contacts/year in Figure 1e.i, corresponding to *R*_0_ = 2.7 in a single population (no vaccination) model), there is no epidemic among the vaccinated in the absence of segregation (*A_v_*(η = 0) = 0). However, a non-zero vaccinated-population attack rate (*A_v_* > 0) occurs if η is sufficiently large, and emerges regardless of whether one isolates or compounds the unvaccinated. Therefore, for small enough values of $c_v^0 = c_u^0$, any segregation could increase infections among the vaccinated.

The main qualitative features of the above results for *P_v_* = 0.8 hold for other values of *P_v_*. Appendix 2 provides a detailed exploration of results for *P_v_* = 0.1 through 0.99; and for two values of *VE* (0.4 and 0.8). When *VE *is decreased, *A_v_* is not strongly influenced by η, regardless of *m_u_*; therefore, any beneficial effect of segregation on *A_v_* is reduced as *VE* decreases.

Appendix 2 also explores $c_v^0 \neq c_u^0$. For example, when $c_v^0 > c_u^0$, the unvaccinated contact frequency is reduced even when there is no segregation; increasing η can then increase *A_v_* substantially compared to the case of $c_v^0 = c_u^0$, holding all other parameter values constant (see panels a.i and b.i in figures A2.28 and A2.31 in Appendix 2).

## Discussion

Segregation can have substantially different and negative impacts on the outcome of an epidemic, depending on the type and degree of segregation, and depending on cultural and population-density factors, for example, that co-determine $c_v^0$ and $c_u^0$.

Segregation that compounds the unvaccinated (*m_u_* > 0 and *m_v_* = 0) generally causes an increase in the vaccinated-population attack rate, *A_v_*, for small and intermediate degrees of segregation, η, while for large η, *A_v_* decreases below its value in an unsegregated society. Segregation that isolates and excludes the unvaccinated (*m_u_* < 0 and *m_v_* = 0) decreases *A_v_* for “more contagious viruses” (i.e. large $c_v^0 = c_u^0$, large *R*_0_); however, for “less contagious viruses” (smaller $c_v^0 = c_u^0$, smaller *R*_0_), both isolating and compounding types of segregation can increase *A_v_* beyond its value in an unsegregated society. For “viruses that are not very contagious” (small $c_v^0 = c_u^0$, small *R*_0_), applying segregation can cause a sizeable epidemic among the vaccinated even though virtually no vaccinated people would be infected in an unsegregated society. Segregation increases the unvaccinated attack rate, *A_u_*, for compounding and moderately isolating types of segregation, and *A_u_* is only decreased for strongly isolating segregation (*m_u_* approaching -1).

Except for large negative values of *m_u_*, and small unvaccinated population fractions, applying segregation has the effect of increasing the frequency of unvaccinated-to-unvaccinated contacts (see figure A1.2 in Appendix 1). This increases the overall probability of a susceptible-infectious interaction, since the unvaccinated population has a higher fraction of susceptibles, and creates a form of core group dynamics [24-26]. At the same time, increasing segregation shields the vaccinated population from the increased prevalence of infection in the unvaccinated population. This trade-off causes the non-monotonic relationship between *A_v_* and η. The same dynamic causes the emergence of an epidemic for large η when $c_v^0 = c_u^0$ (and thus *R*_0_) is small.

We find that *B_v_*, the share of vaccinated infections that are due to contact with unvaccinated people, follows a similar trend to *A_v_* as a function of the degree of segregation, when segregation has no impact on the vaccinated contact frequency (*m_v_* = 0). For this type of segregation, *A_v_* and *B_v_* either increase or decrease simultaneously with increasing η, depending on the value of *m_u_*, and *B_v_* is minimized for complete segregation. When *m_v_* = 0, there is no type or degree of segregation that reduces the vaccinated attack rate while simultaneously increasing the risk to vaccinated people from unvaccinated people (Figure 1). Therefore, there are no circumstances in which the unvaccinated cause a disproportionate risk to the vaccinated, contrary to conclusions in Fisman et al. [9].

In contrast, when *m*_v_ ≠ 0, such that segregation affects the contact frequencies of vaccinated people, increasing segregation can cause *A_v_* to increase while *B_v_* decreases and vice-versa (see figures A2.25 and A2.26 in Appendix 2).

The impact of vaccination-status-based societal segregation on contact frequencies has not previously been considered to our knowledge, even in network-based models in which unvaccinated people cluster together in “cliques” or households [27-29].

Limitations

Our model assumes only two risk populations (vaccinated and unvaccinated), considers only the attack rates on epidemic completion (*A_v_*, *A_u_*, and *A_t_*), and takes the degree of segregation η to be time-independent, without variation due to public holidays and such. It does not consider other outcomes such as death or hospitalization, and does not include different age groups with different characteristics such as contact frequencies or recovery times. Our model assumes an all-or-nothing *VE*, without waning immunity or influence on infectiousness; and no possibility of reinfection. We do not consider the impact of segregation policies on vaccination rates. SIR models and their variations are based on the paradigm of transmission due to pairwise contact between a recently infected and a susceptible individual. However, this paradigm is unable to account for important features of viral respiratory disease incidence; in particular, its rapid emergence and disappearance occurring at essentially the same time at widely dispersed locations [30]. Air-borne transmission via suspended aerosol particles is not directly compatible with pairwise transmission, since it occurs in built environments where many people may transit or be present [31]. A related and unavoidable limitation is the lack of reliable empirical evaluations of needed infectious contact frequencies, which is important because our calculated outcomes are sensitive to the chosen contact frequency values. Lastly, we do not consider the deleterious health impacts of the segregation policies themselves, which can be significant [32-38].

## Conclusions

In the two-population mixing-model framework, vaccination-status-based societal segregation can lead to substantially different and counter-intuitive epidemic outcomes depending on the type and degree of segregation, and depending on complex cultural and physical factors that co‑determine infectious contact frequencies (i.e., the products βc). Negative epidemiological consequences can occur for either segregated group, irrespective of the deleterious health impacts of the policies themselves.

Given the lack of reliable empirical evaluations of needed infectious contact frequency values, the demonstrated outcome sensitivities to the infectious contact frequencies, and the intrinsic limitations of SIR models in this application, we cannot recommend that SIR modelling be used to motivate or justify segregation policies regarding viral respiratory diseases, in the present state of knowledge.

## Figures and Tables

**Figure 2:**
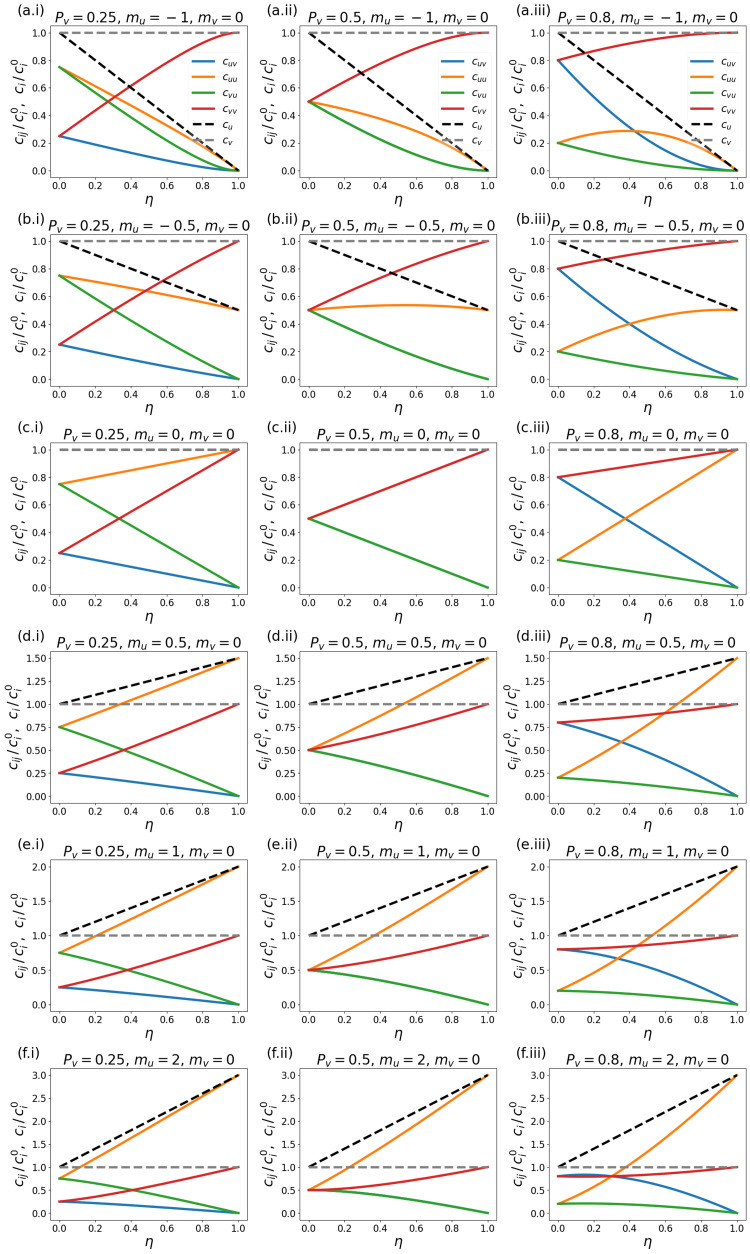
Figure A1.2 from Appendix 1 Normalized population-specific contact frequencies *c_u_* and *c_v_*, intra-population contact frequencies *c_uu_* and *c_vv_*, and inter-population contact frequencies *c_uv_* and *c_vu_*, as functions of η, for the six values of *m_u_* explored in the main text (each row of panels corresponds to a different value of *m_u_*), for *m_v_* = 0, and for *P_v_* = 0.2 (left column of panels), *P_v_* = 0.5 (middle column) and *P_v_* = 0.8 (right column).

**Figure 3:**
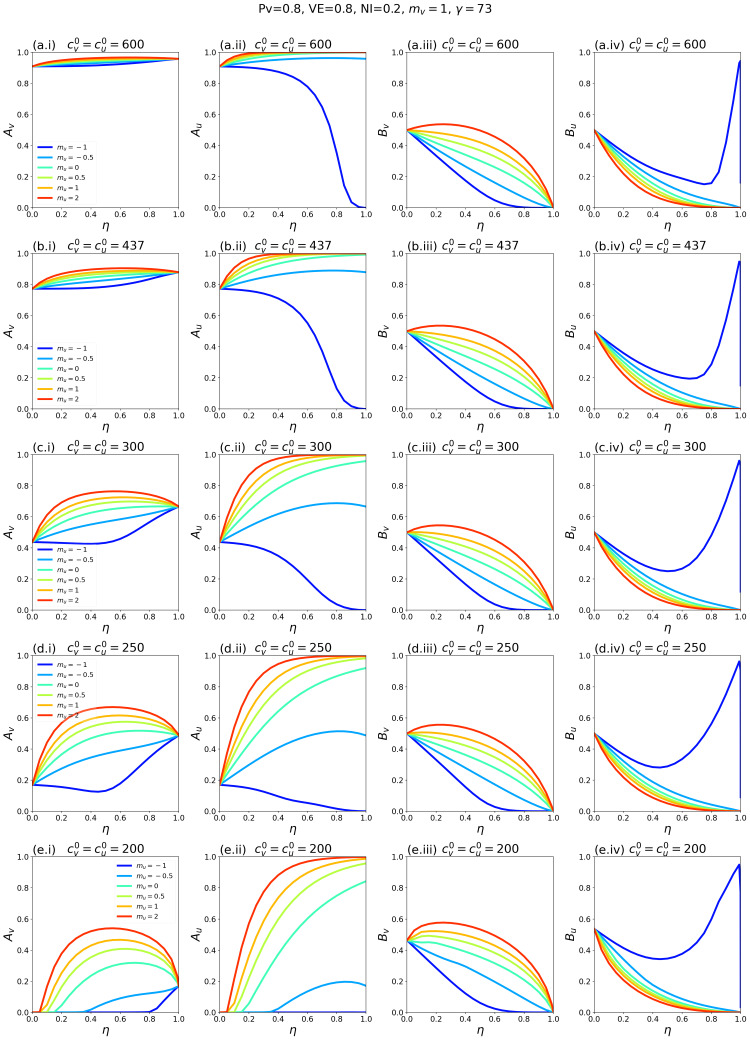
Figure A2.25 from Appendix 2 *A_v_*, *A_u_*, *B_v_*, and *B_u_* and functions of η. Each row of panels corresponds to a single choice of $c_v^0=c_u^0$, and each coloured line to a choice of *m_u_* as indicated in the legends. Parameter values *P_v_*=0.8, *VE*=0.8, *NI*=0.2, *m_v_*=0, *m*_u_ = 1, γ*_v_* = γ*_u_* = 73. This figure is the same as Fig. 1 in the main text, except that *m_v_* = 1.

**Figure 4:**
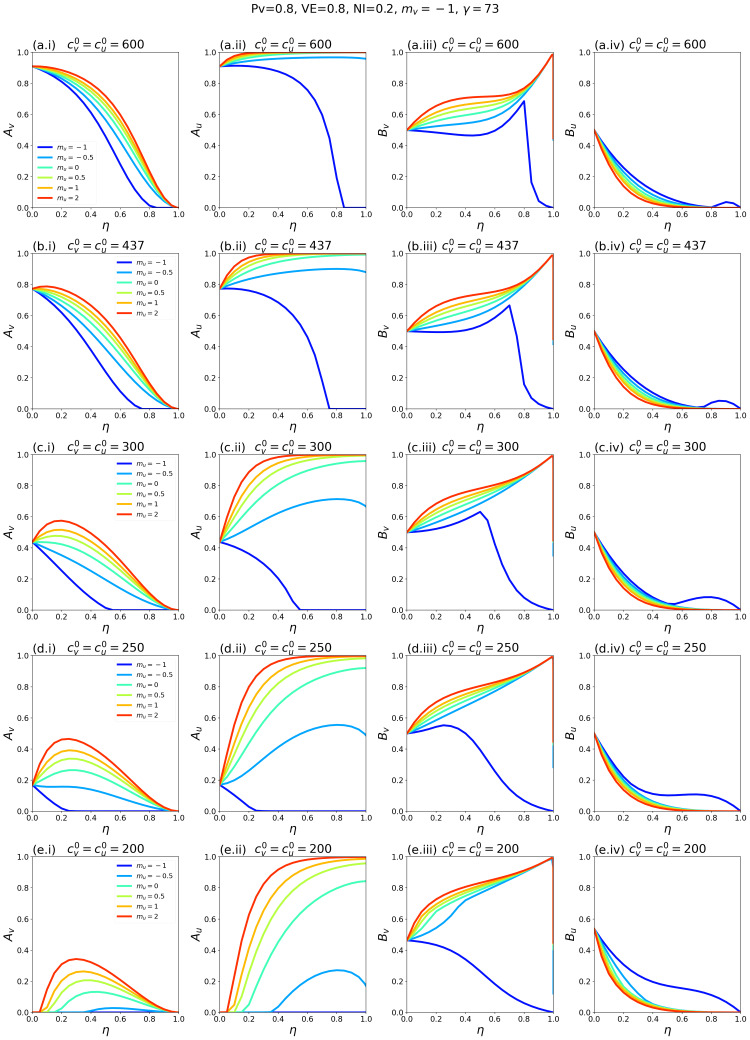
Figure A2.26 from Appendix 2 Same as Fig. A2.25 from Appendix 2, except that *m_v_* = -1.

**Figure 5:**
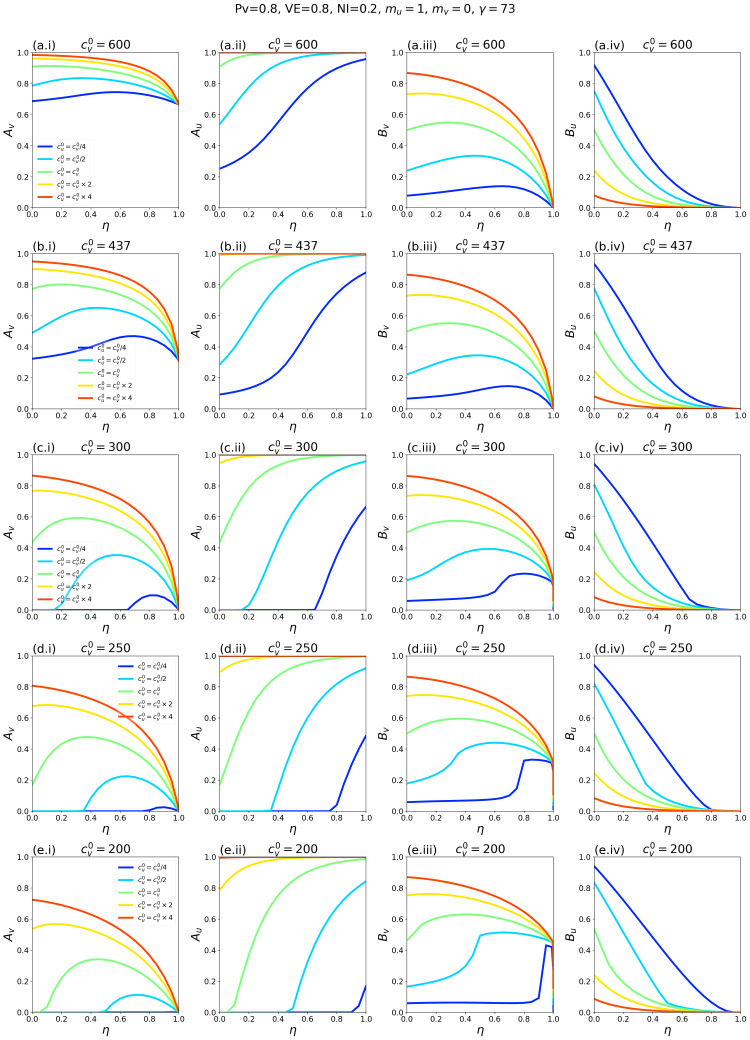
Figure A2.28 from Appendix 2 $c_v^0 \neq c_u^0$, *m_v_* = 0, *m_u_* = 1, *γ_v_* = *γ_u_ *= 73, *VE* = 0.8, *NI* = 0.2, various choices of  $c_v^0/c_u^0$ (see legend within left-column panels), showing *A_v_*, *A_u_*, *B_v_*, and *B_u_* as functions of η. $c_v^0$ is fixed for each row in the figure and decreases moving down the rows.

**Figure 6:**
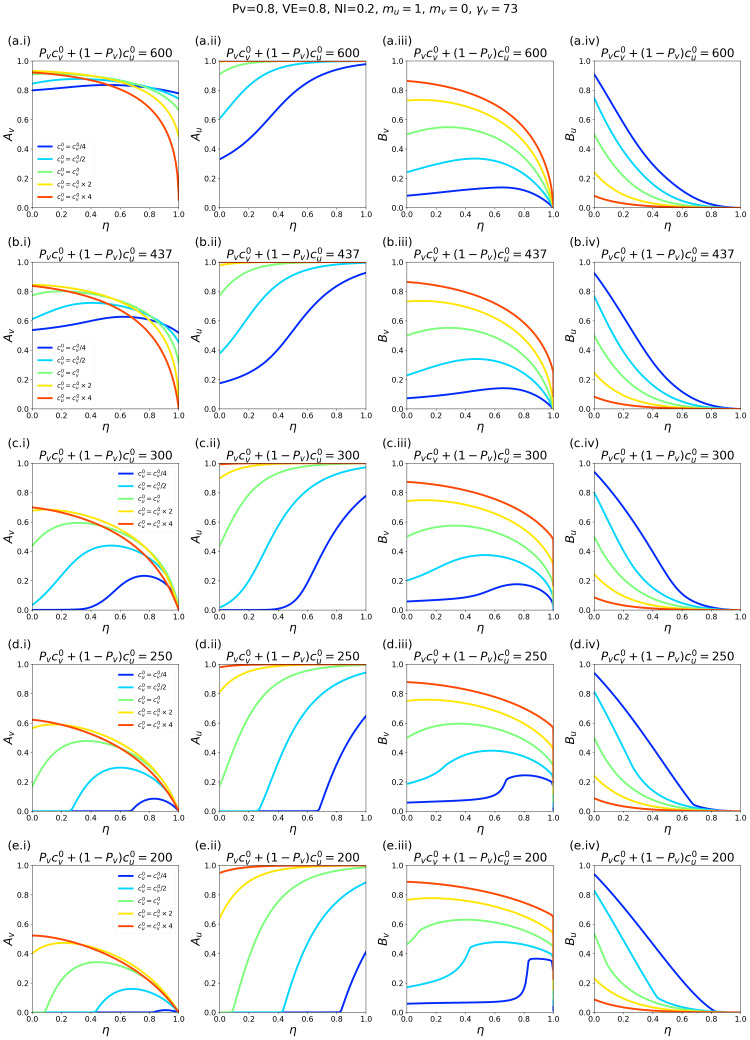
Figure A2.31 from Appendix 2 $c_v^0 \neq c_u^0$, *m_v_* = 0, *m_u_* = 1, γ_v_ = γ_u_ = 73, *VE *= 0.8, *NI *= 0.2, various choices of $c_v^0/c_u^0$ (see legend within left-column panels), showing *A_v_*, *A_u_*, *B_v_*, and *B_u_* as functions of $\eta$. The weighted sum $P_v c_v^0 + (1-P_v)c_u^0$ is fixed for each row and decreases moving down the rows.

## Appendices

Appendix 1

https://figshare.com/articles/preprint/Appendix_1_of_the_pre-print_Compartmental_mixing_models_for_vaccination-status-based_societal_separation_regarding_viral_respiratory_diseases_by_J_Hickey_D_G_Rancourt_posted_to_medRxiv_on_2023-07-19_Version_4_https_www_medrxiv_org_content_1/24431836

Appendix 2

https://figshare.com/articles/preprint/Appendix_2_of_the_pre-print_Compartmental_mixing_models_for_vaccination-status-based_societal_separation_regarding_viral_respiratory_diseases_by_J_Hickey_D_G_Rancourt_posted_to_medRxiv_on_2023-07-19_Version_4_https_www_medrxiv_org_content_1/24431848
